# The clinical and cost-effectiveness of stratified care for patients with sciatica: the SCOPiC randomised controlled trial protocol (ISRCTN75449581)

**DOI:** 10.1186/s12891-017-1513-5

**Published:** 2017-04-26

**Authors:** Nadine E. Foster, Kika Konstantinou, Martyn Lewis, Reuben Ogollah, Kate M. Dunn, Danielle van der Windt, Ruth Beardmore, Majid Artus, Bernadette Bartlam, Jonathan C. Hill, Sue Jowett, Jesse Kigozi, Christian Mallen, Benjamin Saunders, Elaine M. Hay

**Affiliations:** 1 Arthritis Research UK Primary Care Centre, Research Institute for Primary Care & Health Sciences, Keele University, Staffordshire, ST5 5BG UK; 2 Keele Clinical Trials Unit, Keele University, Staffordshire, ST5 5BG UK; 3 Health Economics Unit Institute of Applied Health Research, University of Birmingham, Edgbaston, Birmingham, B15 2TT UK

**Keywords:** Stratified care, Sciatica, Primary care, Randomised controlled trial

## Abstract

**Background:**

Sciatica has a substantial impact on patients, and is associated with high healthcare and societal costs. Although there is variation in the clinical management of sciatica, the current model of care usually involves an initial period of ‘wait and see’ for most patients, with simple measures of advice and analgesia, followed by conservative and/or more invasive interventions if symptoms fail to resolve. A model of care is needed that does not over-treat those with a good prognosis yet identifies patients who do need more intensive treatment to help with symptoms, and return to everyday function including work. The aim of the SCOPiC trial (**SC**iatica **O**utcomes in **P**r**i**mary **C**are) is to establish whether stratified care based on subgrouping using a combination of prognostic and clinical information, with matched care pathways, is more effective than non-stratified care, for improving time to symptom resolution in patients consulting with sciatica in primary care. We will also assess the impact of stratified care on service delivery and evaluate its cost-effectiveness compared to non-stratified care.

**Methods/Design:**

Multicentre, pragmatic, parallel arm randomised trial, with internal pilot, cost-effectiveness analysis and embedded qualitative study. We will recruit 470 adult patients with sciatica from general practices in England and Wales, over 24 months. Patients will be randomised to stratified care or non-stratified care, and treated in physiotherapy and spinal specialist services, in participating NHS services. The primary outcome is time to first resolution of sciatica symptoms, measured on a 6-point ordered categorical scale, collected using text messaging. Secondary outcomes include physical function, pain intensity, quality of life, work loss, healthcare use and satisfaction with treatment, and will be collected using postal questionnaires at 4 and 12-month follow-up. Semi-structured qualitative interviews with a subsample of participants and clinicians will explore the acceptability of stratified care.

**Discussion:**

This paper presents the details of the rationale, design and processes of the SCOPiC trial. Results from this trial will contribute to the evidence base for management of patients with sciatica consulting in primary care.

**Trial registration:**

ISRCTN75449581, date: 20.11.2014.

## Background

About 60% of patients with low back pain (LBP) report pain in the leg (s) [1] although not all will be diagnosed as having sciatica. Sciatica is a common variation of LBP presenting with radiating pain in the leg and often accompanied by variable neurological changes in sensation, reflex or muscle strength in the leg [2]. The most common reason for sciatica symptoms is a disc prolapse compressing or irritating a spinal nerve root [2]. Sciatica prevalence estimates vary widely from 1.2 to 43%, a variation that is related to using different diagnostic criteria and sampling methods [3]. It is believed that many patients with sciatica have a favourable outcome and experience resolution of symptoms within 12 weeks from onset [2, 4]. However, a substantial proportion (estimated at up to 30%) continues to suffer with pain for a year or more [2]. Studies from primary and secondary care show that recovery after one year is moderate, varying from 49 to 58%, depending on the definition of recovery [5, 6]. The literature indicates that compared to LBP alone, sciatica has a more substantial impact on patients, with longer pain episodes [7], it is also responsible for a large proportion of the indirect costs and days lost from work associated with LBP. A Dutch study estimated that the cost of sciatica to society represents 13% of all LBP related costs [8], which translates to an annual impact to the UK economy of £268 million in direct medical costs and £1.9 billion in indirect costs.

The evidence for treatments for sciatica has been summarised in several systematic reviews [9–12]. Overall they highlight the poor quality of the research to date, mostly small trials limited to short**-**term follow**-**up. The results of the reviews indicate that the efficacy and tolerability of drugs commonly prescribed in primary care for sciatica (such as non**-**steroidal anti-inflammatory drugs, corticosteroids, antidepressants, anticonvulsants, muscle relaxants and opioid analgesics) is unclear [11]. There is evidence that active physiotherapy increases the proportion of sciatica patients showing improvement and is especially effective for those with severe symptoms [13, 14]. The reviews reach conflicting conclusions about the role of spinal injections, although these appear to provide pain relief in the short**-**term [15, 16]. Surgery provides more rapid recovery from the symptoms of sciatica although outcomes are similar to those from non**-**surgical care, one or two years later [4, 17]. Surgery and spinal injections are associated with more frequent and more severe adverse events [10], optimal selection criteria for eligible patients for surgery (discectomy) are lacking [18], and immediate referral to surgery for all sciatica patients is not a cost-effective model of care [10].

In practical terms, current treatments range from providing information and advice, medications, exercise, traction, acupuncture and manual therapy, to more invasive treatments such as spinal injections and surgery. Although there is variation between clinicians, generally the current model of care followed for sciatica is ‘stepped’. This typically means that initially there is a ‘wait and see’ period in primary care with advice and pain medication, then for those patients not improving after a period of weeks or months, referral to a clinician such as a physiotherapist might be considered, for treatments including exercise and manual therapy. Subsequently, patients failing to improve might be referred to specialist spinal services for investigations and further management [2]. Currently the only patients who are fast**-**tracked from primary care to spinal specialist opinion are those with suspected cauda equina syndrome or profound, widespread or progressive neurological deficit, and those who need hospitalisation due to pain severity. There are no robust estimates for the proportion of patients consulting in UK primary care with sciatica who proceed to spinal injection or spinal surgery, although some old reports estimate that between 5**-**15% of patients with sciatica proceed to disc surgery [18, 19]. Approximately 13% of patients consulting with sciatica in UK primary care are referred to spinal specialist services at some point following their initial primary care consultation [20]. Currently, there is no evidence that can robustly guide decision making about which sciatica patients to refer early for consideration of interventions such as spinal injections and/or surgery.

The UK Spinal Taskforce [21] highlighted problems in the management of sciatica and emphasised the urgent need for good quality trial evidence to underpin treatment decision-making, including better information about the clinical and cost effectiveness of early referral of patients with severe symptoms, for consideration of secondary care treatments such as surgery or spinal injections. A model of care is needed that does not over-treat those with a good prognosis, yet promptly identifies the patients who do need more active treatment to help with symptoms and return to everyday function including work [22].

A model of stratified care that uses information on the risk of persistent disabling non-specific LBP (prognostic risk: low, medium, high) and targets treatment accordingly, has been shown to be superior to non-stratified primary care [23, 24]. The approach uses a brief self-report tool - the STarT Back tool [25] which was developed for, and validated with, primary care patients with non-specific LBP (with and without leg pain). A similar model of stratified care may be beneficial for sciatica patients consulting in primary care, but evidence is lacking.

## Trial aims and objectives

The overall aim is to investigate whether the management of adult patients with sciatica presenting in primary care can be improved through a model of stratified care. The primary objective is to compare the clinical effectiveness of stratified care to non-stratified care, in terms of patient**-**reported time to resolution of sciatica symptoms.

Secondary objectives are to: a) compare the clinical effectiveness of stratified care to non**-**stratified care, on a range of important outcomes, including physical function, pain, quality of life, work loss, healthcare use and satisfaction with treatment, b) compare the cost-effectiveness of stratified care compared to non**-**stratified care, c) investigate the impact of stratified care on service delivery, specifically the proportion of patients receiving stratification-appropriate referrals and treatments, and d) determine the acceptability of aspects of the stratified care model, to patients and clinicians.

## Methods

### Design

The SCOPiC trial is a multicentre, pragmatic, assessor**-**blind, two-arm randomised controlled trial (RCT), comparing stratified care versus non-stratified care for adults with sciatica, with internal pilot and concurrent health economic evaluation and linked qualitative study.

### Setting

Participants will be recruited from approximately 30 general practices in Staffordshire, North Shropshire, Cheshire, and Wales, UK. The population in these localities is a mix of urban, inner city, semi-rural and rural. The trial involves National Health Service (NHS) general practices, NHS physiotherapy services and NHS spinal specialist services. A number of research clinics (SCOPiC sciatica clinics), in which patients are screened for eligibility and recruited to the trial, are based in primary care centres, in Staffordshire, North Shropshire (patients from general practices in Wales are seen in North Shropshire), and Cheshire.

### Participants

Participants are eligible for inclusion if they are aged 18 years and over, consulting at their general practice with back and/or leg symptoms of any duration or severity and their general practitioner (GP) (or other healthcare practitioner (HCP) in the practice) suspects sciatica, are able to communicate in English, willing to participate, able to give full written consent, have access to a mobile phone or landline, and following clinical assessment in the SCOPiC research clinic have the diagnosis of sciatica confirmed.

Exclusion criteria are: potentially serious spinal pathology (such as cauda equina syndrome, malignancy, inflammatory spondyloarthopathy), previous lumbar surgery, pregnancy, serious physical or mental co**-**morbidity preventing them from attending the research clinic and/or undergoing assessment and interventions, currently receiving ongoing care from, or have been in consultation with, a secondary care doctor or physiotherapist for the same problem in the last 3 months, are currently participating in any other sciatica research study.

### Stratified care model for sciatica

#### Stratification algorithm

We devised an algorithm to identify those sciatica patients likely to need a fast-track referral from primary care to specialist spinal services, and those who are likely to do well with treatments available in primary care. This algorithm was based on data analysis from the ATLAS study cohort [20] which provided information on the characteristics of sciatica patients most likely to be referred to spinal specialist services. Full details of the development of the stratification algorithm will be provided in a separate paper. Here we give very brief details on the stratification algorithm and the three derived subgroups. We utilised prognostic information, using the STarT Back tool [25], and clinical information based on the following clinical examination findings: interference with ability to work (including work around the house), pain below the knee, intense leg pain, and sensory changes in the painful leg during neurological examination (loss of or reduced pin prick sensation in a dermatomal distribution in the painful leg). The algorithm allocates patients to one of three subgroups, and each subgroup is matched with a care pathway. We allocated patients to **subgroup 1**, which involves referral to primary care for management options of low treatment intensity, if their total score on the STarT Back tool was less than or equal to 3 out of a possible 9. We used a combination of the STarT Back tool score and a number of findings from the clinical examination described above to direct referral to primary care management options of higher treatment intensity (**subgroup 2**), or to fast-track patients to a specialist spinal opinion and imaging tests (**subgroup 3**). The details of the care pathways for each of the three subgroups, are presented in full later in the text, under the section ‘Stratified care arm: Matched care pathways and their delivery’.

## Recruitment procedures

### Identification and invitation of potentially eligible patients

Participants are identified when they consult their GP (or other healthcare practitioner (HCP) within the practice) with symptoms of sciatica. When a patient with back and/or leg pain consults, and an appropriate Read code [26] is entered on the computer system, a ‘pop-up’ prompt screen asks the GP if he/she thinks the patient might have sciatica, and if so, to consider whether the patient is suitable to be invited to the SCOPiC sciatica clinic, taking into account trial inclusion/exclusion criteria. Entering ‘yes’ (that the patient has possible sciatica and is suitable to be invited to the SCOPiC sciatica clinic) on the computer system flags those patients thought to be suitable for invitation to the clinic and allows the GP to briefly inform the patient about the clinic and the trial. Using the same ‘pop up’ screen the GP also is asked to record what their management plan would have been for the patient outside the trial (‘continue with GP care’, ‘physiotherapy referral’, ‘specialist spinal referral’). This information will be summarised at the end of the trial in order to describe GP management preferences at first consultation (e.g. continue with GP care, refer to physiotherapy services or refer to spinal specialist services).

On a weekly basis, letters inviting patients to the SCOPiC sciatica clinic are mailed to all potentially eligible patients identified from their consultation. The letter and clinic information sheet explains that there is a research study being hosted at the SCOPiC sciatica clinic. Patients are invited to telephone the clinic administrator to make an appointment at the SCOPiC sciatica clinic. During that telephone call, the administrator carries out a brief check for suitability for the clinic. Patients who are receiving or have received care from alternative or complementary HCP practitioners such as osteopaths, chiropractors, acupuncturists or others, are not be excluded but are advised, where possible, to keep co-interventions to a minimum during the treatment phase of the trial. The administrator offers a clinic appointment within 10 working days. A letter is sent to patients to confirm their appointment details, together with a participant information sheet and a baseline questionnaire. Approximately two days before the clinic appointment, a clinic administrator telephones patients to remind them about their appointment and ask those who are interested in taking part in the research to bring their completed questionnaire.

Weekly retrospective GP practice consultation records review may also be used, if needed, as a second method of identifying potentially eligible participants. With this method there is a search for patients for whom the GP has entered one of the agreed Read codes indicating he/she suspects sciatica, so that if GPs overlook or do not have time to complete the electronic ‘pop up’ screen in the consultation, this method will ensure these patients are identified retrospectively and also invited using the same procedures described above. Duplication checks will ensure that the same patient is not invited twice.

### Full eligibility screening and informed consent

The SCOPiC sciatica clinics operate as integrated research/service clinics. Physiotherapists will explain the purpose of the clinic, and answer any questions patients have about the clinic or the trial. Patients expressing interest in participating in the trial proceed to have a standardised assessment for sciatica by the physiotherapist, to establish full eligibility and ascertain subgroup allocation. Eligibility for the trial is based on the assessing physiotherapist being ≥70% confident of a diagnosis of sciatica [27]. Patients who have leg pain thought by the assessing physiotherapist to be due to causes other than sciatica are excluded (for example: referred leg pain, hip pathology, peripheral neuropathy, vascular pain), as are patients for whom there is substantial diagnostic uncertainty. Patients included in the trial will have pain in one or both legs, plus at least one of the following self-reported symptoms or clinical findings; leg pain approximating a dermatomal distribution, leg pain worse or as bad as the back pain, leg pain made worse by coughing/sneezing/straining, subjective sensory symptoms approximating a dermatomal distribution, any degree of objective neurological findings relating to spinal nerve root (s) involvement such as sensory or reflex changes or myotomal weakness, or positive neural tension tests (straight leg raise (SLR), femoral nerve stretch). The above symptoms and signs are part of the presentation of sciatica [28, 29].

**Fig. 1:**
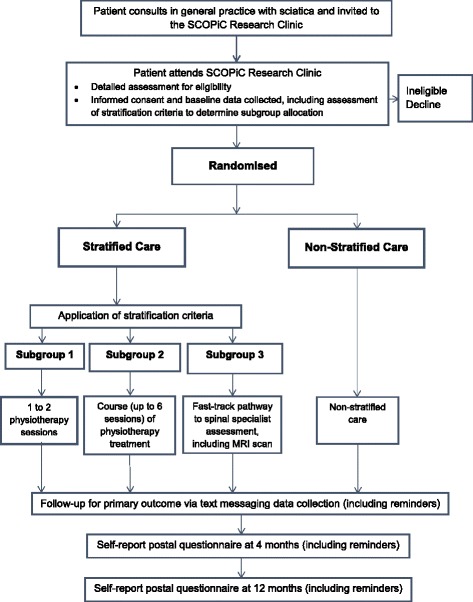
Summary flow diagram of participant recruitment in the SCOPiC trial

For patients who are eligible and interested in taking part in the trial, the physiotherapist explains the trial in detail, answer any questions and, if they are willing to participate, takes written informed consent. Patients who are ineligible or who do not wish to participate will receive advice and education from the physiotherapist in clinic and then continue with care as appropriate, outside the trial. Figure 1 outlines the SCOPiC recruitment procedures.

### Randomisation and allocation concealment

Eligible patients who consent to take part are randomised to one of the two trial arms using a 1:1 web-based randomisation service through Keele Clinical Trials Unit (CTU), ensuring allocation concealment. The clinic administrator uses the web-based randomisation method (with a back-up process to telephone the CTU for randomisation in the event of web access failure), providing details of the centre (Staffordshire, North Shropshire/Wales, Cheshire) and patient subgroup allocation. Individual patients are randomised, stratified by centre and subgroup allocation, using random permuted blocks of varying size, to either stratified care or non**-**stratified care. The administrator informs the physiotherapist of the patient’s allocation (stratified care arm or control arm) and delivery of treatment will commence within the same SCOPiC clinic visit. The patient’s GP is informed in writing that the patient is participating in the trial but are not informed about which arm of the trial the patient has been randomised to. Usual clinician to clinician correspondence continues as per normal practice and the research does not interfere with this.

### Blinding and protection against bias

Selection bias at recruitment is avoided by separating the processes of determining patient eligibility and treatment allocation and by using random permuted blocks overseen by the CTU, not allowing physiotherapists assessing and treating patients to predict the next allocation in the clinic. Patients are told that the trial is comparing two primary care approaches for the treatment of sciatica, one based on matching patients to treatment using a simple tool which helps to decide on the treatment pathway most likely to help them, and one based on treatment needed as agreed by the physiotherapist in clinic and themselves. Patients randomised to non**-**stratified care are seen by physiotherapists who have not carried out their detailed clinical assessment and eligibility screen for the trial, and thus the treating physiotherapist in the control arm will remain blind both to the details of the stratification algorithm and the individual patient’s subgroup status, in order to make sure that there is no contamination between the two trial arms. It is not possible to blind physiotherapists treating participants randomised to the stratified care treatment arm, therefore, in order to further protect against contamination, no physiotherapists treating patients in the stratified care arm are involved in the treatment of any patients randomised to the control arm. Research nurses blinded to treatment allocation conduct primary outcome data collection over the telephone (for participants not using the automated text messages) and minimum data collection (secondary outcomes) over the telephone at 4 and 12-months follow-up, for patients who do not respond to questionnaires. The risk of contamination from GPs knowing to which arm a patient is randomised is very low. However, we will check whether involvement in the trial changes GPs’ referral habits for this condition by increasing GPs’ awareness about sciatica. We will check this by describing the proportion of sciatica patients referred to other services from participating general practices for the period of 12 months prior to the start of the trial and for the duration of the trial recruitment period. Using anonymised GP electronic records, we will retrospectively check referral patterns as described above. In qualitative interviews with the GPs we will also collect data on whether involvement in the trial contributed to changes in GPs’ referral decisions for patients with sciatica. The trial databases are password protected to ensure that the research nurses and trial statisticians remain blind to treatment allocation. Anonymised data comparisons between consenting and non-consenting individuals, dropouts and completers will be carried out to evaluate external validity and risk of attrition bias. Additionally, using validated outcome measures with established reliability will reduce measurement error.

### Audit of interventions

Treatment delivered in the intervention arm of the SCOPiC trial is recorded in a standardised format on Case Report Forms (CRFs). This includes, date of start and completion of treatment, number of treatments received and types of interventions (i.e. exercise, advice, manual therapy). Protocol deviations are reported and recorded. Details of the care patients receive in the ‘fast-track’ pathway and timeframe of any interventions delivered (such as surgical or injection procedures) are collected by populating CRFs with the relevant information from the clinical letters generated in the specialist clinics for each patient. Following the 12-month follow-up, reviews of hospital medical records for the patients in the ‘fast-track’ pathway for information on the sciatica treatments they have accessed will also be undertaken for completeness.

All physiotherapists who deliver care to patients in the control arm of the SCOPiC trial also record treatment details on a CRF. Similarly, this includes the dates of the start and completion of treatment, number of treatments and types of interventions received. Physiotherapy record reviews are conducted in the cases of missing or incomplete CRFs for both stratified care and control participants.

## Internal pilot

The internal pilot phase of the SCOPiC trial will assess recruitment and follow-up rates over the first 8 months of recruitment, success of GP practice recruitment and retention, success of physiotherapy site recruitment including training and engagement, adherence to the treatment protocols, suitability of the patient selection criteria, proportion of participants allocated to each of the three subgroups according to the stratification algorithm, time to magnetic resonance imaging scan (MRI) and specialist opinion for those in the fast-track pathway (subgroup 3), the event rate of the primary outcome, and rate of missing data for the primary outcome up to 4-month follow-up for all participants recruited within the 8 months of the pilot trial phase. Details of the internal pilot analysis are described under the ‘Statistical analyses’ section.

## Stratified care arm: Matched care pathways and the delivery of treatments

### Physiotherapy management in primary care

#### Subgroup 1 (STarT Back tool; Low risk)

Patients in this subgroup are expected to have a good prognosis and will receive a brief treatment package delivered by trial physiotherapists. In contrast to patients with non-specific LBP and a score of 3 or less on the STarT Back tool, patients with sciatica tend to have more severe symptoms, hence the decision to offer a referral to physiotherapy for brief treatment. Patients receive up to two 30-min sessions with the physiotherapist, with a target of delivery over 4 weeks, in order to permit review where needed but no further sessions will be offered. The package of care is tailored to the individual patient’s presentation and specific needs in order to support self-management and reduce disability. It includes advice, information, appropriate reassurance and education about sciatica. It focuses on the expected good prognosis and recovery without the need for further tests or investigations, the maintenance of activity levels including return to work where appropriate, and lifestyle advice such as general activity and weight control as appropriate as well as guidance on self-management and management of future flare-ups of sciatica. Pain relief and appropriate medication are also discussed with any suggested changes in analgesia communicated to the patient and their GP for consideration. To reinforce key messages, a sciatica booklet (developed from existing educational materials already available in the sciatica literature) will be given to the patient along with an information sheet of local contacts for exercise venues such as swimming pools, exercise classes and physical activity opportunities.

#### Subgroup 2

Patients allocated to this subgroup receive a course of physiotherapy treatment, tailored to their individual needs. This matched treatment package is delivered in an initial 45-min session with a target of up to 6 further 30-min sessions over 6 to 12 weeks. The STarT Back tool score and clinical assessment findings guide the treating physiotherapist in targeting management towards the physical and psychosocial factors that are particular problems for each patient. The physiotherapist agrees an individualised treatment plan with the patient according to their need and best current evidence. The main aims of treatment are to reduce pain, decrease disability and address modifiable physical and psychological obstacles to recovery. Management plans include some or all of the following: advice, explanation, reassurance and education, medication review and advice (with any suggestions on analgesia communicated to the patient and their GP for consideration), exercise (McKenzie ‘directional preference exercises’, strengthening-muscle stability exercises, general fitness and mobility exercises and guidance on pacing using graded activity principles depending on patient presentation), manual therapy techniques (joint and/or soft tissue), acupuncture and advice about and plans for return to normal activities and work as appropriate. Psychological obstacles to recovery, such as fear-avoidance beliefs, pain related low mood and distress or anxiety, unhelpful or erroneous beliefs about back pain and sciatica and catastrophising are also addressed as part of the physiotherapy treatment. The same sciatica booklet and information sheet of local contacts for exercise venues, as those given to patients in subgroup 1, are also given to patients in subgroup 2. Management of future flare-ups of sciatica are addressed as part of the treatment plan. Consistent with evidence based guidelines, bed rest, traction, massage and electrotherapy are not included in the treatment options [10].

The treatments for subgroups 1 and 2, are delivered in primary care by NHS physiotherapists. Participants continue to be able to access other care via their GP. The physiotherapists treating patients in subgroups 1 and 2 are responsible for providing good clinical governance to their patients and will be permitted to overrule the stratification algorithm recommendation for matched care pathways, if they strongly believe this is necessary. As in our previous trials^23^, such protocol deviations are expected to be rare and will always be discussed with the treating physiotherapist’s spinal specialist mentor and the trial principal investigator (PI), and documented.

### Fast-track care pathway to specialist spinal service

#### Subgroup 3

Patients allocated to subgroup 3 are fast**-**tracked to specialist assessment and opinion about suitability for other treatments, such as spinal injections or surgery. ‘Fast**-**track’ is defined as immediate referral from the SCOPiC sciatica clinic to specialist spinal assessment with service level agreements in place with participating NHS services to ensure that assessment will take place within 4 weeks of the patient’s SCOPiC sciatica clinic appointment. An MRI scan is part of the specialist spinal assessment for the patients in the ‘fast**-**track’ care pathway. A report on the MRI scan will be provided by a consultant radiologist, as per normal NHS clinical practice. The spinal specialist has access to the MRI results, as part of their assessment of patients in this ‘fast**-**track’ pathway. It is important to note that the ‘fast**-**track’ pathway is to *specialist assessment and opinion* and not to surgery or injection. Patients with contraindications to MRI see the spinal specialist who will decide on alternative imaging tests as necessary. The specialist, in discussion with the patient, determines the most appropriate treatment based on assessment and MRI findings and patient preference.

These spinal specialist services within the participating NHS services, include specialist clinics at the primary/secondary care interface (usually delivered by extended scope spinal physiotherapy specialists), spinal orthopaedic and pain clinic teams. The first appointment with specialists for patients allocated to the ‘fast-track’ pathway is at the primary / secondary care interface. These NHS services are delivered by specialist spinal physiotherapists (extended scope practitioners), any referrals to other spinal specialist services (orthopaedics, neurosurgery or pain clinic) are decided as part of routine care at this point.

#### Non-stratified care and its delivery

The control arm of the SCOPiC trial is based on non-stratified primary care, delivered by physiotherapists. Patients randomised to the control arm are treated by a physiotherapist in the SCOPiC sciatica clinic. For these patients, treatment includes a one**-**off session (in the same clinic visit) of advice and education. The treating physiotherapist then decides whether the patient should be discharged back to the care of their GP or be referred for community physiotherapy or to specialist spinal services. If an onward referral is required this is arranged by the physiotherapist at the clinic.

As previously mentioned, in order to avoid contamination between the stratified care arm and control arm, at the SCOPiC clinic, different physiotherapists provide treatment for patients in each of the two trial arms and the physiotherapists who are providing treatment to control care patients are unaware of the details of the stratification algorithm and individual participants’ group status.

#### Training for participating physiotherapists

Participating physiotherapists will attend training workshops with the trial team prior to the start of patient recruitment and treatment. Those delivering the one-off treatment for patients in the control arm in the SCOPiC clinic, will take part in a half-day workshop about trial procedures, importance of avoiding contamination between trial arms and the completion of trial CRFs.

Those involved in the stratified care arm of the trial will attend three days of training. The focus of the training will be on carrying out the standardised assessment according to agreed protocols to identify patients with sciatica for participation in the trial, the stratification algorithm, taking informed consent, the delivery of evidence based physiotherapy interventions in line with the biopsychosocial model of care and the procedures of the trial, avoiding contamination between trial arms, and the completion of trial CRFs.

The training will be supplemented by comprehensive written material on the trial procedures, and on guidelines and treatment algorithms for the evidence based assessment and treatment of patients with sciatica. To maximise protocol fidelity, physiotherapists treating patients in subgroups 1 and 2, will have support as required, this will be provided by the research team’s spinal physiotherapy specialists from the spinal services participating in the trial.

## Outcome measures and data collection

### Primary outcome

The primary outcome measure is time to first resolution of symptoms of sciatica, measured on a 6-point ordered categorical scale: ‘completely recovered’, ‘much better’, ‘better’, ‘same/ no change’, ‘worse’ and ‘much worse’ – the anchor being against the patients’ baseline symptoms when they attended the SCOPiC sciatica clinic (“compared to how you were at the SCOPiC clinic X weeks/months ago, how are your back and leg symptoms today?”). Patient-reported resolution of symptoms is defined as a response of either ‘completely recovered’ or ‘much better’, collected using regular text messages (or brief phone calls where text messaging is not possible). Data collection for the primary outcome occurs weekly, starting on the first Sunday following the participant’s assessment at the SCOPiC sciatica clinic, for the first 4 months for all participants. Then between 4 and 12-month follow-up, the text message data collection changes to once every 4 weeks, or until ‘stable resolution’ of symptoms, which is defined as 2 consecutive months’ responses of ‘completely recovered’ or ‘much better’. After stable resolution, data collection for the primary outcome via text message ceases. 94% of UK adults have a mobile phone (https://www.ofcom.org.uk./facts) and previous research has shown that weekly text messages are a useful method of data collection to examine the clinical course of back pain in primary care, with high mean response rate of 83% [30]. Participants who do not respond to their first week’s text message receive a reminder message 48 h later, and those who still do not respond are mailed a postcard the next day. Participants who do not respond to the second week’s text message receive a reminder message 48 h later, and those who still do not respond receive a phone call from a research nurse after at least a further 24 h. For subsequent non-response the reminder processes described is repeated. If the participant continues not to provide a response using text message there is an option to transfer to data collection by brief phone call.

### Secondary outcomes

Secondary clinical outcomes evaluate health status at 4 and 12 months using participant self-completed postal questionnaires with postal reminders and minimum data collection over the telephone, by research nurses. A text message is sent to participants at 4 and 12-month follow-up to notify them that the SCOPiC follow-up questionnaire will soon arrive in the post. Measures include Global Perceived Change ((GPC) (6-point Likert scale as per the primary outcome data collection)), physical function limitations (sciatica version of the Roland and Morris Disability Questionnaire) [31], overall impact of sciatica symptoms (Sciatica Bothersomeness Index) [32], pain intensity of back and leg pain [33], sleep disturbance (Jenkins sleep questionnaire) [34], fear of movement (Tampa Scale of Kinesiophobia) [35], anxiety and depression (Hospital Anxiety and Depression Scale) [36], risk of poor outcome according to the STarT Back tool [25], health related quality of life (EQ-5D-5L) [37], general health (SF-1) [38], neuropathic pain using the S-LANSS (self-report Leeds Assessment of Neuropathic Symptoms and Signs) [39], days lost from work and productivity loss due to sciatica, pain medication, adverse events and satisfaction with care received and its results. See Table 1 presents an overview of procedures and measures used according to the SPIRIT statement (Chan et al. [40]).

**Table 1:**
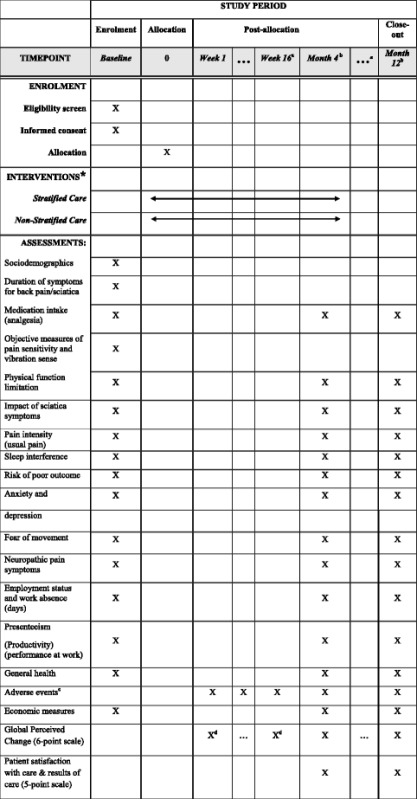
Overview of enrolment, interventions, and assessments

*The interventions and their delivery are described in detail in the manuscript. Treatments are tailored to the individual participant and likely to have different frequency and duration for each participant. Overall, treatment for most participant is expected to be completed within 4 months from randomization

^**a**^ The trial’s primary outcome is time to symptoms resolution. Data collection with text messages for the primary outcome occurs weekly for the first 4 months for all participants. Between 4 and 12-month follow-up, the text message data collection changes to once every 4 weeks, or until ‘stable resolution’ of symptoms, which is defined as 2 consecutive months’ responses of ‘completely recovered’ or ‘much better’. After stable resolution, data collection for the primary outcome via text message ceases

^**b**^ At 4 and 12 months follow-up, data collection is via postal questionnaires

^**c**^ Participants and clinicians are asked to report any adverse and/or serious adverse events. Patients are asked about adverse events in the follow-up questionnaires

^**d**^ Global perceived change is collected via text messages or telephone calls, and in the follow-up questionnaires at 4 and 12 months

### Process outcomes

Process outcomes are collected to investigate the impact of stratified care on service delivery. Numbers and proportions of patients in each arm of the trial receiving appropriate referrals and treatments according to their subgroup allocation, and the timing of starting treatment, are collected from CRFs, patient questionnaires and record reviews from participating NHS services.

### Health economic outcomes

Health economic outcomes are collected to determine the costs of the interventions (stratified care and control) and other sciatica-specific healthcare utilisation. Resource use information is obtained on primary care consultations (GPs and other HCPs in the practice), secondary care consultations (e.g. hospital consultants), prescriptions, hospital based procedures (diagnostic tests, injections), nature and length of inpatient stays, surgery and over-the-counter purchases by patients. Patients are asked to distinguish between UK NHS and private provision. Cost data are collected via participant questionnaires at 4 and 12 months. If hospital records data on sciatica-related surgery or injections (to supplement patient-reported data) can be obtained, this data will be included in a sensitivity analysis. Unit costs are obtained from standard sources including the British National Formulary (BNF) [41], Unit Costs of Health and Social Care [42] and NHS Reference costs. Information is also collected from participants about their occupational status, sciatica-related time off work and reduced work performance (presenteeism) [43], to enable the calculation of productivity costs, allowing analysis from a societal cost perspective. The average wage for each respondent will be identified using UK Standard Occupational Classification coding [44] and annual earnings data for each job type [45]. The analysis will use the human capital approach, and the self-reported days of absence will be multiplied by the respondent-specific wage rate. The outcome of interest for the economic analysis is quality-adjusted life years (QALYs) and these will be calculated using EQ-5D-5L responses obtained at baseline, 4 and 12 months.

## Adverse events

Information is collected on adverse events (AEs) experienced by trial participants and potentially related to trial interventions or procedures. Physiotherapists treating patients in the SCOPiC trial are asked to report potential AEs by the use of CRFs or report to the trial team. Patients are asked about AEs in the follow-up questionnaires. Expected AEs include for example a transient increase in pain as a result of a new exercise programme. Any serious adverse events (SAEs), defined as an event that is life threatening, results in death, unscheduled hospitalisation, or significant disability, is immediately reported to the trial team according to the procedures described in the Keele CTU standard operating procedures for SAEs. Any SAEs considered to be related to the trial procedures or interventions will be reported to the main Research Ethics Committee by the Chief Investigator within 15 days of becoming aware of the event. In addition, all such events will be reported to the trial sponsor, Trial Steering Committee (TSC) and Data Monitoring Committee (DMC).

## Qualitative study

The aim of the linked qualitative study is to understand the acceptability of the ‘fast-track’ pathway in the stratified care arm of the trial, to patients and clinicians. Qualitative research on patients’ experiences of sciatica and its management is scarce [46, 47], especially in those with most severe symptoms, and qualitative studies that report clinicians’ views of managing this condition are even less common [48]. In the SCOPiC trial stratified care arm the ‘fast-track’ pathway to spinal specialist assessment and opinion is novel, and the focus of the qualitative study is on patients in this subgroup and the clinicians involved in their management.

A purposive sample of patients randomised to the stratified care arm and who are on the ‘fast-track’ pathway will be invited for interview based on treatment centre and patient demographics including age, gender, baseline leg pain severity/disability, and also response to treatment. Interviews will be conducted after the 4 month follow-up point.

Potential interviewees will be sent an invitation letter with an information leaflet about the interview, following which a researcher will contact them by phone, and arrangements made with those who agree to participate. Consent will be obtained at the start of the interview, either in writing if the interview is face-to-face, or audio-recorded if the interview is over the telephone. A semi-structured topic guide will be developed, exploring patients’ views of their clinical care, its appropriateness and timeliness for addressing their problem, and the time taken to assessment, treatment (s) and symptom resolution. Patients’ experience of sciatica symptoms and their impact will also be explored to provide context in terms of treatments received. Participating spinal specialist clinicians (spinal physiotherapy specialists, spinal surgeons), and a sample of participating GPs will be invited for interview after the trial finishes participant recruitment. Again, using a semi-structured topic guide, the interviews will explore clinicians’ views about the acceptability of the ‘fast-track’ pathway, the suitability of patients on this pathway, and explore how the ‘fast-track’ pathway compares with usual care, what the added value of the ‘fast-track’ pathway might be for this subgroup of sciatica patients, and what, if any, limitations there might be. In interviews with GPs we will also explore their perceptions of whether their involvement in the trial contributed to changes in their referral decisions for patients with sciatica. The same invitation and consent process as described above for patients will be followed with the clinicians. We estimate that sufficient data saturation [49] will be reached with a sample of 20 to 25 patients’ and clinicians’ interviews (total of approx. 40 to 50 interviews).

## Statistical analyses

The trial analysis will be conducted and reported following the Consolidated Standards of Reporting Trials (CONSORT) guidelines [50–52].

### Clinical outcomes analysis

#### Primary analysis

The primary analysis will compare time to self-reported resolution between stratified care and non-stratified care, on an intention-to-treat basis. A Kaplan-Meier survival analysis will estimate the time from randomisation until first resolution of symptoms. Participants who drop out of the trial through active withdrawal will be censored at the time this occurs (by contrast, participants not responding at any time point will continue to be followed-up until they actively withdraw). This will provide the data for comparing the relative mean and median survival times of the two trial arms. A Cox proportional hazards regression analysis will compare time to resolution between arms by calculation of a hazard ratio (HR) of rates of resolution adjusted for centre, patient stratification group (subgroups: 1, 2, 3) and baseline pain duration. The HR will be presented along with a 95% confidence interval for the HR; a p-value < 0.05 (two-tailed) based on the Wald test statistic will signify rejection of the null hypothesis of no difference in recovery time between the two arms. A statistically significant p-value with HR > 1 would indicate statistically significant shorter time to recovery for stratified care compared to control; a statistically significant p-value with HR < 1 would indicate a (statistical) significantly longer time to recovery for stratified care compared to control. The analysis will account for physiotherapist effect (proportional Cox hazards frailty model).

#### Sensitivity analyses (of the primary endpoint)

The following sensitivity analyses will be carried out to test the rigour and robustness of the primary outcome evaluation through evaluation of:

- alternative assumptions regarding missing data – for the primary evaluation, missing data are assumed to be synonymous with non-recovery. The primary evaluation is based on ‘recovery’ at the first point of a positive response, but if any missing data immediately precede this response then all these observations are assumed to be indicative of ‘non-recovery’ – an additional sensitivity analysis will set the time interval of recovery as the mean time between the participant’s last response (indicating ‘non-resolution’) and the time at which ‘resolution’ is first classified. A more extreme sensitivity analysis will take the contrary view on missingness to equate to, and imputed as, a ‘resolved’ case.
- alternative assumption regarding interval censoring – to account for the fact that only the interval of time within which the resolution occurred is known, and not the exact time (especially after the first 16 weeks when outcome data is collected monthly). A further sensitivity analysis will use methods that allow for interval censoring.
- secondary definitions of good outcome - we will use three separate secondary classifications based on ‘stable resolution’, defined as: two consecutive recordings of ‘completely recovered’ or ‘much better’; ‘improvement’, defined as: completely recovered’, ‘much better’ or ‘better’, through single response, and ‘stable improvement’, defined as: ‘completely recovered’, ‘much better’ or ‘better’, through two consecutive responses.
- analysis of participants in the stratified care arm that: (i) have complete follow-up – i.e. not including censoring, and (ii) receive the treatment pathway that is correctly matched to their subgroup – as per treatment protocol.

#### Exploratory subgroup analyses

A small number of re-analyses of the primary endpoint analysis of time to resolution will be carried out to include testing the effectiveness of stratified care for those with/without suspected disc-related radicular pain as determined by clinical assessment, and for patients in each of the stratification subgroups (subgroups 1, 2 and 3). Descriptive statistical summaries will be provided through mean/median time to resolution per treatment arm and per patient subgroup (subgroups 1, 2, 3) according to suspected/not suspected disc-related radicular pain. Tests of statistical significance will be through evaluation of 95% interval estimates / p-values for the interaction term for the product of group variable (with/without suspected disc-related radicular pain) by treatment arm within the Cox regression model adjusting for centre, stratification group (stratifying variables), and pain duration.

#### Secondary outcomes analysis

The analysis will provide between-arm differences in secondary outcomes at 4 and 12 months and provide point and 95% interval estimates from longitudinal linear and logistic mixed effect regression models as appropriate to the outcome data being analysed adjusting for centre, stratification subgroup (1, 2, 3) (stratifying variables) and baseline pain duration. Descriptive summary of mean scores for the two trial arms and difference in mean scores (numerical outcomes) and frequency counts (percentages) along with odds ratios (categorical outcomes) will be presented, with between-arm comparisons being presented in the form of point and 95% interval estimates, alongside p-values for the test of statistical association.

#### Assumption checking

The Cox regression model assumption of proportional hazards will be examined in two ways: (i) firstly, through graphical review of the survival curves – if the survival curves are observed to cross this would indicate non-proportionality; (ii) through inclusion of a time-arm interaction in the regression model with statistical significance of this term signifying important deviation from the assumption of proportional hazards. If either of the two examinations shows violation of the proportional hazards assumption then alternative statistical testing will be performed using an unadjusted log rank test.

For the linear models we will examine inverse normal plots to check for normality (in the event of any reasonable violation we will use a suitable data-transformation function). Potential covariance structures will be explored for goodness-of-fit of mixed models through comparing likelihood and Bayesian Information Criteria (BIC).

#### Process outcomes analysis

Descriptive statistics will be used to examine healthcare resource utilisation by stratification subgroup (subgroups 1, 2, 3) and the number and percentage of participants who proceeded to secondary care treatments, and the types of interventions they received, in both trial arms – and by stratification subgroup (1, 2, 3). Non-parametric tests will be used to test for between-arm comparisons.

#### Internal pilot analysis

Analysis of the internal pilot data will provide information on success of GP practice and physiotherapy site recruitment and retention, recruitment and follow-up rates (over the first 8 months of recruitment), adherence to the treatment protocols, proportion of participants allocated to each of the three subgroups (1, 2, 3), time to ‘fast-track’ MRI and specialist opinion for those in subgroup 3 (‘fast-track’ pathway), the event rate of the primary outcome (recovery) up to 4-month follow-up for all participants (stratified care and control arms) recruited within the 8-month pilot phase in order to check the sample size calculation assumptions. The rate of missing data for the primary outcome will also be checked in the pilot phase for all participants. No formal interim analysis of participants’ clinical outcomes is proposed for the internal pilot.

## Sample size

We have calculated a sample size of 470 participants in total, in order to test for superiority of stratified care compared to non-stratified care. The primary outcome of interest in the SCOPiC trial is time to resolution of symptoms (defined as patient self-report of being ‘completely recovered’ or ‘much better’ compared to baseline (from the 6-point ordinal GPC scale). In our previous trial of stratified care for LBP [23], nearly 60% of patients in total had a clinically important improvement on the GPC scale at 4 and/or 12-month follow up; the absolute difference between arms of that trial at 4 months was 11%. If proportional hazards are to be assumed (i.e. in this case assumed relative rate of ‘resolution’), this difference would equate to an HR in the interval 1.4 - 1.5. Allowing for 20% dropout, a sample size of 470 (235 per treatment arm) is required to detect an HR between 1.4 - 1.5 with 80-90% power (given a two-tailed significance level of 5%), assuming a rate of resolution in excess of 60%, and intra-class correlation (ICC) for physiotherapist effect less than 0.01 (based on estimates from other primary care trials [23, 53], and allowing for a coefficient of variation in physiotherapist cluster size of 0.65 [54]; specifically:

- an HR of 1.4 in median survival times with 90% power (if all participants in the trial are recovered by 12-month follow-up and ICC for therapist effect is <0.001) (least conservative)
- an HR of 1.5 in median survival times with 80% power (if 60-65% of participants in the trial are recovered by 12-month follow-up and ICC for physiotherapist effect is 0.01) (most conservative)

An HR above 1 (and correspondingly lower ‘survival’ function) in this context is a positive result in contrast to traditional survival analysis of mortality.

This sample size will also provide more than 80% power to detect a ‘small’ to ‘moderate’ standardised mean difference (effect size) of 0.35 [55] between the two trial arms in a key secondary outcome of physical disability at 12-month follow-up, allowing for an ICC for physiotherapist effect of 0.01.

## Economic analysis

The within**-**trial health economic analysis will determine the cost**-**effectiveness of stratified care compared with non**-**stratified care. An incremental cost**-**utility analysis will be undertaken using participants responses to the EQ**-**5D**-**5L questionnaire to calculate the cost per QALY gained. The base**-**case analysis will adopt a health care perspective, incorporating UK NHS and private sciatica-related healthcare resources utilised during the 12-month follow-up period. Analysis from a wider societal perspective will explore the impact on the results when productivity costs are taken into account. Additional exploratory analyses will consider the cost**-**effectiveness of stratified care compared with non-stratified care for participants in each subgroup (subgroups 1, 2 and 3 separately), a strategy used previously in studies of stratified care for LBP [56, 57]. Deterministic and probabilistic sensitivity analyses will be conducted to test the robustness of the results and overall uncertainty in the trial cost and outcome data respectively. Cost-utility planes and acceptability curves will be derived in order to provide a graphical display (plane and curve) and quantification (curve) of the level of uncertainty around incremental cost-effectiveness ratios (ICERs).

## Qualitative analysis

Audio recordings of interviews will be transcribed verbatim, checked and anonymised. Data will be managed and shared using NVivo software, fully coded and analysed thematically [58]. To ensure sufficient data saturation, analysis will follow an iterative process, with topic guides being revised in light of emergent themes. There will be a clearly documented and on-going process of detailed coding, both within and across cases searching for confirmatory and contradictory findings [59]. Data from the patient interviews will also be compared for differences and similarities with the outcome data from the trial [60]. Emerging themes will be explored and discussed at regular trial team meetings which will include clinicians and social scientists. In addition, early findings from the qualitative interviews will be presented to our research user group, which includes individuals who have experienced sciatica symptoms, and their views of the data incorporated into further data collection and analysis.

## Trial organisation and monitoring

The trial is sponsored by Keele University. The day to day operation of the trial will be overseen by the Trial Management Group (TMG) and all trial procedures will adhere to Keele CTU standard operating procedures. The trial is monitored by an independent TSC with expertise in LBP, sciatica and clinical trials. An independent DMC is tasked with monitoring patient safety and trial data integrity. During the trial period through to 12-month follow-up, no interim analyses are planned, unless judged necessary by the DMC. The TSC, DMC, TMG and clinicians involved will remain unaware of the trial results until all data are cleaned, checked and analysed after the 12-month data are collected.

### Data confidentiality and archiving

All trial-related information will be stored securely at Keele CTU, Keele University. Data will be anonymised using coded identification numbers and the data and the linking code will be stored in separate locations, under password protection. Access to the data will be to the small number of individuals necessary for quality control, audit and analysis. We will publish and communicate the trial results regardless of the outcome of the trial. Data from the SCOPiC trial will be archived and made available for future, secondary analysis and data pooling purposes, by the SCOPiC team or other research teams.

### Ethics

The trial received ethical approval by the National Research Ethics Service (NRES) Committee West Midlands - Solihull (17/03/15): Project Reference Number: 15/WM/0078. Site-specific approvals have been received from the appropriate local R&D offices. The trial is being conducted in accordance with the ethical principles in the Declaration of Helsinki and good practice guidelines on the proper conduct of research.

## Patient and Public Involvement

We previously interviewed patients with sciatica and they highlighted its impact, the need for clearer information on treatment and prognosis and patients’ willingness to balance their desire for pain relief with adverse effects [46]. Whilst planning the SCOPiC trial we held a workshop with three patient representatives (who currently had sciatica or had suffered with it in the past) from a research user group. This highlighted the importance of early assessment and diagnosis and the need to get patients to treatments that match their problem more quickly. They all felt that early pain relief is the key outcome, given the severity of the pain and that regular, brief, text messages or phone calls that collect data about symptoms, were acceptable. Two patient representatives are members of the TSC for the SCOPiC trial. Members of a wider research user group advised on the study documents, the language of the text messaging and the interpretation of data from the qualitative interviews with patients.

## Discussion

This paper presents the rationale for, and the design and processes of the SCOPiC trial. The trial is investigating the clinical and cost-effectiveness of stratified care (subgrouping patients and matching them to one of three care pathways) for patients consulting in primary care with symptoms of sciatica. The novelty of this trial is threefold. First is the use of information about a patient’s risk of persistent physical disability due to the pain, and findings from the clinical examination, in order to allocate patients to one of three subgroups. Second is the matched care pathways. Subgroups 1 and 2 are expected to do well with conservative treatment in the form of physiotherapy, with patients in subgroup 1 receiving up to two brief physiotherapy sessions, and patients in subgroup 2 receiving a course of up to six physiotherapy sessions. Patients in subgroup 3 are those more likely to need a referral to specialist services and are ‘fast-tracked’ to imaging tests and specialist spinal services for an opinion on management. The third novel feature is the choice of primary outcome, time to symptom resolution assessed regularly using text messages.

The results of the SCOPiC trial will inform patients, clinicians, service commissioners, and future clinical guidelines, as to the clinical and cost-effectiveness of stratified care for sciatica. We anticipate participant recruitment will be completed in 2017, and 12-month follow-up data collection will be completed in 2018.

### Trial status

Recruitment is ongoing. Results are expected in 2018.

## Abbreviations

AEs: Adverse events
BIC: Bayesian information criteria
BNF: British national formulary
CONSORT: Consolidated standards of reporting trials
CRF: Case report form
CTU: Clinical trials unit
DMC: Data monitoring committee
GP: General practitioner
GPC: Global perceived change
HCP: Health care practitioner
HR: Hazard ratio
ICC: Intra-class correlation
ICERs: Incremental cost-effectiveness ratios
LBP: Low back pain
MRI: Magnetic resonance imaging
NHS: National health service
NRES: National research ethics service
QALYs: Quality-adjusted life years
RCT: Randomised clinical trial
SAEs: Serious adverse events
SCOPiC: SCiatica Outcomes in Primary Care
SLR: Straight leg raise
TMG: Trial management group
TSC: Trial steering committee
